# Optimized CAP cut-offs for metabolic dysfunction associated steatotic liver disease in patients living with obesity: a large biopsy-based prospective study

**DOI:** 10.1038/s41598-026-47209-y

**Published:** 2026-04-20

**Authors:** Riham Soliman, Mohamed Elbasiony, Ahmed Helmy, Nabiel Mikhail, Helmy Ezzat, Ahmed Mehrez Gad, Ebrahim Abdel Halim, Khaled Zalata, Rokia Masoud, Ayman Hassan, Ahmed Farahat, Mohamed El Emam, Gamal Shiha

**Affiliations:** 1 Technical Institute of Nursing, Sherbin, Mansoura Egypt; 2 Egyptian Liver Research Institute and Hospital (ELRIAH), Sherbin, Mansoura Egypt; 3 Gastroenterology and Hepatology Unit, Internal Medicine Department, Faculty of Medicine, Mansoura University, Mansoura, Egypt; 4 Tropical Medicine and Gastroenterology Department, Faculty of Medicine, Assiut University, Assiut, Egypt; 5 Biostatistics and Cancer Epidemiology Department, South Egypt Cancer Institute, Assiut University, Assiut, Egypt; 6 Gastrointestinal surgery center, Faculty of Medicine, Mansoura University, Mansoura, Egypt; 7 General Surgery Department, Faculty of Medicine, Delta University for Science and Technology, Mansoura, Egypt; 8 Surgery Department, Faculty of Medicine, Horus University, New Damietta, Egypt; 9 Pathology Department, Faculty of Medicine, Mansoura University, Mansoura, Egypt; 10 Higher Technological Institute of Applied Health Science, Sherbin, Mansoura Egypt; 11 Internal Medicine Department, Faculty of Medicine, Egyptian Liver Research Institute and Hospital (ELRIAH), Mansoura University, Mansoura, Egypt

**Keywords:** Controlled attenuation parameter (CAP), Non-invasive diagnosis, Steatosis, Steatohepatitis, MASLD, Liver biopsy, Diseases, Gastroenterology, Medical research

## Abstract

Controlled Attenuation Parameter (CAP) quantifies hepatic steatosis non-invasively, but current cut-offs limit diagnostic precision, especially in obese populations. We aimed to validate CAP against liver biopsy and propose optimized thresholds tailored for obesity. In this single-centre, cross-sectional, biopsy-validated study, 798 adults undergoing laparoscopic cholecystectomy (*n* = 629) or sleeve gastrectomy (*n* = 169) between 2019 and 2024 were included. All underwent Fibro Scan and wedge liver biopsy. Histology was scored by two blinded pathologists. Diagnostic accuracy of CAP was assessed using AUROC, sensitivity, specificity, predictive values, calibration, net reclassification improvement (NRI), integrated discrimination improvement (IDI), and decision curve analysis (DCA). Newly derived cut-offs were benchmarked against existing ones. Histology revealed S0 in 72.7%, S1 in 19.7%, S2 in 7.0%, and S3 in 0.6%. Standard CAP cut-offs showed suboptimal specificity (49.7% for ≥S1, 55.8% for ≥S2). Newly derived thresholds of 290 dB/m (≥ S1) and 317 dB/m (≥ S2) improved specificity (74.4% and 82.5%) while maintaining good sensitivity (62.7% and 71.7%). NRI and DCA demonstrated superior alignment and higher net benefit compared with all published thresholds. Optimized CAP cut-offs (290 and 317 dB/m) provide better diagnostic precision and clinical utility than current thresholds strengthening MASLD screening and clinical trial eligibility, particularly in obese populations.

**Supplementary Information:**

The online version contains supplementary material available at 10.1038/s41598-026-47209-y.

## Introduction

Fatty liver disease affects approximately 25% of the global population and has become a leading cause of chronic liver disease^1^. The prevalence of hepatic steatosis has increased in parallel with the global rise in obesity and metabolic syndrome^2^. Moreover, more than 50% of individuals with metabolic disorders are affected by steatosis, which is increasingly recognized as a significant contributor to the development of cirrhosis and hepatocellular carcinoma (HCC)^3–5^. Notably, HCC can develop even in patients without cirrhosis, underscoring the clinical importance of early detection and management^6^.

The term *Metabolic dysfunction-associated steatotic liver disease (MASLD)* has recently been proposed to replace the previous nomenclature for fatty liver diseases. The diagnostic criteria require the presence of hepatic steatosis in combination with one or more of the following: overweight or obesity, type 2 diabetes mellitus, or evidence of metabolic dysregulation^7^. Liver biopsy remains the reference standard for evaluating steatosis grade^8^. However, liver biopsy has several limitations, including invasiveness, sampling error, and only moderate intraobserver and interobserver reproducibility^9^. These limitations preclude the feasibility of using liver biopsy as a repeated measure to assess histological changes.

Several non-invasive, quantitative, and objective methods for evaluating hepatic steatosis have been developed^10,11^. While conventional B-mode ultrasonography can detect steatosis, its subjective interpretation and inability to quantify liver fat limit its clinical use^12^. Thus, objective, examiner-independent methods such as MRI-derived proton density fat fraction (MRI-PDFF) have emerged to provide more accurate quantification of liver fat however it is not widely available^13–15^.

The Controlled Attenuation Parameter (CAP) is a physical measure based on ultrasonic signals acquired by FibroScan, quantifying ultrasound attenuation at the central frequency of the vibration-controlled transient elastography (VCTE) at the M probe^16,17^. However, the systematic review and meta-analysis by Pu K. et al. (2019) identified significant heterogeneity in the sensitivity and specificity of CAP across different studies from diverse locations and populations^18^. Furthermore, the systematic review and meta-analysis conducted by Shi K. et al. (2014), which included 9 studies involving 1,297 patients with various grades of biopsy-proven steatosis from Europe, Asia, and the USA across multiple centers, demonstrated that CAP values had limited accuracy for diagnosing steatosis. The authors advised caution in the clinical application of CAP^19^. Finally, a meta-analysis by Wang Y. et al. (2015) highlighted the need for additional studies with larger sample sizes to better establish the accuracy of CAP for clinical use^20^.

Recently, two drugs—Semaglutide and Resmetirom—have been approved for the treatment of steatotic liver disease. Their use depends on an accurate diagnosis of MASLD, often guided by CAP measurements^21,22^. Therefore, there is an urgent unmet need to refine CAP cut-offs to minimize overdiagnosis, avoid unnecessary treatment, and ensure appropriate patient selection.

Our objective was to assess the diagnostic accuracy of the currently used cut off CAP against histological steatosis grading and to propose optimized cut-offs tailored to obese individuals.

## Patients and methods

### Study design and reporting standards

**Fig. 1:**
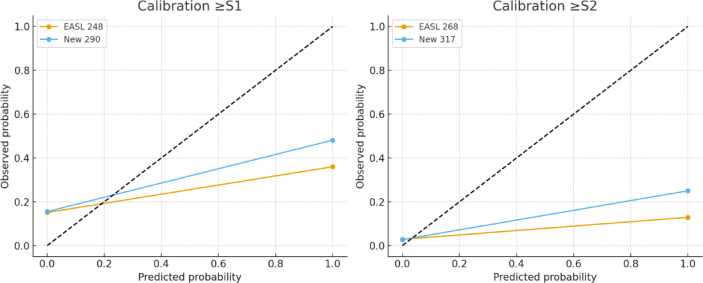
Calibration plots. Calibration plots comparing observed versus predicted probabilities of steatosis using standard EASL cut-offs and newly proposed thresholds. Hosmer–Lemeshow statistics showed good model calibration for both ≥S1 and ≥S2 steatosis.

**Fig. 2:**
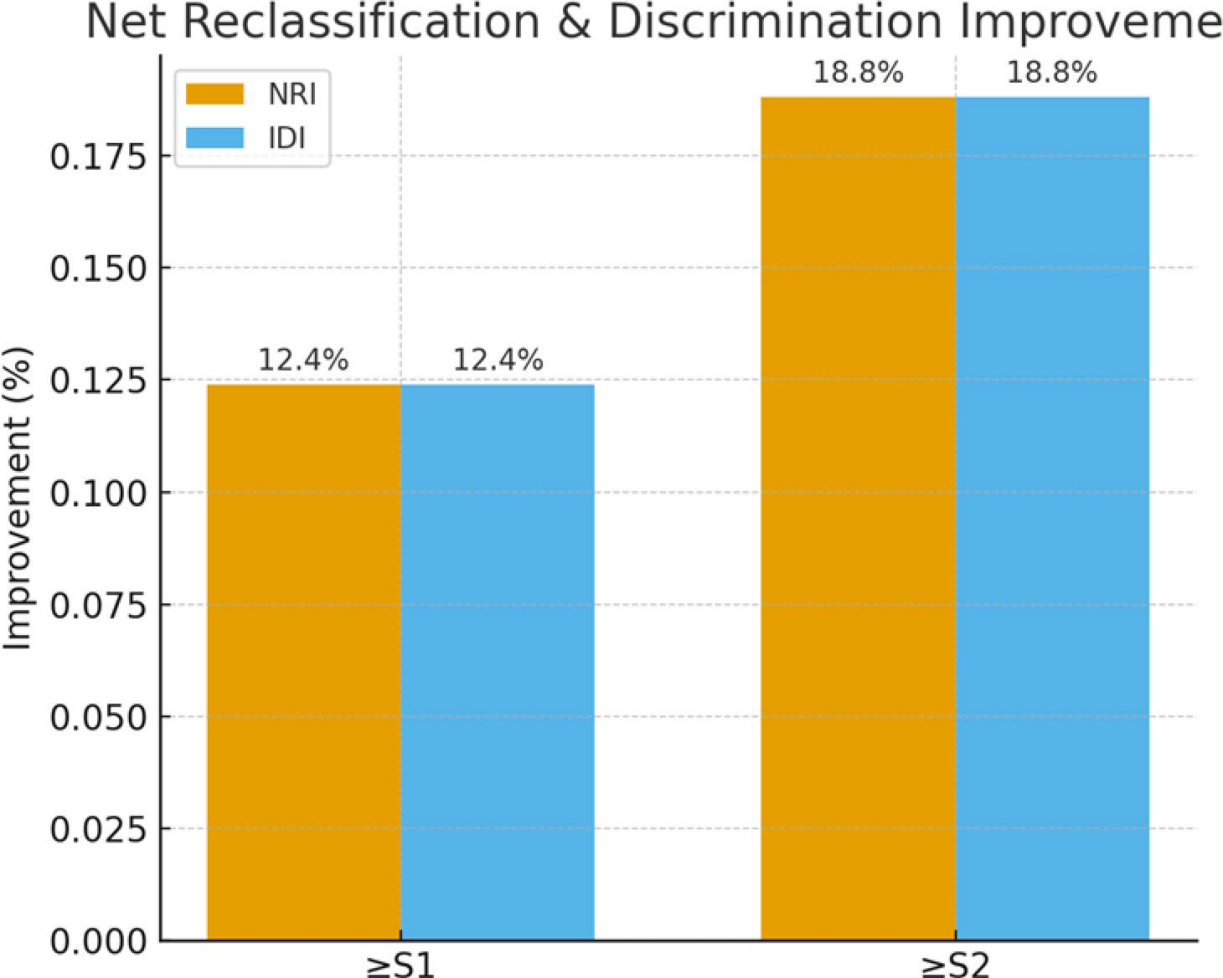
Net Reclassification Improvement (NRI) and Integrated Discrimination Improvement (IDI). Bar plots displaying overall NRI and IDI values when comparing proposed CAP thresholds (290 dB/m for ≥S1, 317 dB/m for ≥S2) against standard EASL thresholds (248 and 268 dB/m). Results show marked gains in non-event reclassification (improved specificity) with modest trade-offs in event reclassification (sensitivity).

This diagnostic accuracy, cross-sectional, prospective, single center study was conducted in accordance with the Standards for Reporting Diagnostic Accuracy Studies (STARD) criteria 2015^23^ to ensure transparency and completeness in reporting. The STARD checklist was used to guide the design and reporting of the study 0 (Fig 1 and 2).

### Study approvals and consent

This study followed the Declaration of Helsinki, its 2008 revisions, and the International Conference on Harmonization recommendations on Good Clinical Practice. The ELRIAH Institutional Research Board (IRB00010534) approved the protocol. Each patient signed a written informed consent form.

### Study participants

This study was conducted between January 2019 and May 2024 on 798 consecutive patients receiving laparoscopic cholecystectomy (*n* = 629) or sleeve gastrectomy (*n* = 169) at the Egyptian Liver Research Institute and Hospital (ELRIAH), Sherbin, Mansoura, Egypt. Patients aged ≥ 18 years, capable of providing written informed consent, and scheduled for liver biopsy during their scheduled surgery were eligible for inclusion. Participants were tested negative for hepatitis B surface antigen, and hepatitis C virus RNA.

Patients were excluded if they had ascites, previous liver transplantation, significant cardiopulmonary disease, active malignancies. A flowchart of patients’ selection is shown in Figure S1**.**

### Fibro scan CAP and LSM assessment

FibroScan examination was performed by physicians trained and certified by the manufacturer and blinded to the patient’s histological evaluation. CAP and liver stiffness measurements (LSM) were obtained using VCTE technology. simultaneously by the FibroScan 502 device (Echosens, Paris, France). CAP measures were performed with two experienced operators within 2–3 weeks prior to surgery, using the XL probe for patients with body mass index (BMI) ≥ 30 kg/m²​​. Measurements were considered reliable if ≥ 10 valid readings were obtained, with an interquartile range < 30% of the median value and a success rate ≥ 60%^24,25^.

All CAP measurements were obtained using the XL probe; therefore, the proposed cut-offs apply specifically to XL-probe assessments and are not intended for direct use with M-probe measurements.

### Liver biopsy procedure

A wedge biopsy of the liver was performed during laparoscopic surgery using two standard laparoscopic bowel graspers to secure hepatic tissue. At an approximately 90-degree angle to create a wedge of hepatic tissue for biopsy. A specimen was excised using laparoscopic scissors, and the cut surface was coagulated with a monopolar device​​. This technique is rapid, safe, inexpensive and requires no specific instruments or devices.

### Histopathologic evaluation

Laparoscopic liver biopsy is obtained during laparoscopic cholecystectomy or laparoscopic sleeve gastrectomy. An adequate liver biopsy specimen was defined as a specimen that incorporates at least 20 mm core tissue length or at least 11 portal tracts^26^. Two experienced pathologists independently reviewed the biopsies. Interobserver variability was quantified using Cohen’s kappa statistic (κ = 0.82), indicating strong agreement, with consensus reached in cases of discrepancy. The pathologists were blinded to each other’s reading and to patients’ clinical and FibroScan data. Fibrosis was staged on a 0–4 scale (METAVIR): F0, no fibrosis; F1, portal fibrosis without septa; F2, portal fibrosis and few septa; F3, numerous septa without cirrhosis; and F4, cirrhosis^27^. Steatosis was scored on 0–3 scale: grade 0, < 5%; grade 1, 5%-33%; grade 2, > 33%-66%; and grade 3, > 66%^12^.

### Statistical analysis

Only patients with histology data and accessible median LSM or CAP values who had at least 10 reliable measurements have been analyzed. Additionally, no attempt has been made to replace any missing data. All analyses were performed using the Statistical Package for Social Sciences version 26 (SPSS, IBM Corp., USA).

Diagnostic accuracy of CAP was assessed by calculating sensitivity, specificity, positive predictive value (PPV), negative predictive value (NPV), and the area under the receiver operating characteristic curve (AUROC) for the detection of ≥S1, ≥S2, and S3 steatosis. Optimal cut-off values were determined using the Youden index. Calibration, net reclassification improvement (NRI), and integrated discrimination improvement (IDI) were used to evaluate the incremental value of the new thresholds compared with existing ones. To assess clinical utility, we performed decision curve analysis (DCA) across a plausible range of threshold probabilities (0.05–0.60). Net benefit of our new cut-offs was compared against “treat-all,” “treat-none,” and published cut-offs. Analyses were conducted on the entire study cohort with complete paired CAP and histology data^28–31^.

## Results

### Patients’ characteristics

A total of 798 patients were included in the study, with a median age of 40 years (IQR: 32–48). The cohort consisted of 154 males (19.3%) and 644 females (80.7%). Among these, 629 patients (78.8%) underwent laparoscopic cholecystectomy, and 169 patients (21.2%) underwent laparoscopic sleeve gastrectomy. The median body mass index (BMI) was significantly higher in the laparoscopic sleeve gastrectomy group (43.9 kg/m², IQR: 34.7–50.1) compared to the laparoscopic cholecystectomy group (33.4 kg/m², IQR: 29.4–38.7; *p* < 0.001; Table 1). Patients in the laparoscopic sleeve gastrectomy group were younger (median age: 36 years, IQR: 29–43.5) than those in the laparoscopic cholecystectomy group (median age: 41 years, IQR: 33–50; *p* < 0.001). CAP values were higher in the laparoscopic sleeve gastrectomy group (median: 298 dB/m, IQR: 225–342) compared to the laparoscopic cholecystectomy group (median: 258 dB/m, IQR: 213–301), *p* < 0.001).

**Table 1:** Characteristics of all patients included (*n* = 798), and as per type of operation.

Variable	All patients *n* = 798 (100%)	Laparoscopic cholecystectomy (LC) *n* = 629 (78.8%)	Laparoscopic sleeve gastrectomy (LSG) *n* = 169 (21.2%)	*P* value
Age (years)	40 (32–48)	41 (33–50)	36 (29-43.5)	< 0.001
Sex Male Female	154 (19.3) 644 (80.7)	117 (18.6) 512 (81.4)	37 (21.9) 132 (78.1)	0.336
BMI (kg/m^2^)	34.9 (29.8–41.2)	33.4 (29.4–38.7)	43.9 (34.7–50.1)	< 0.001
ALT (IU/L)	18 (13.4–26.5)	18.1 (14–27)	16.1 (12.1–24.0)	0.015
AST (IU/L)	18 (15–22)	18.0 (15.0–22.0)	16.5 (13.1–21.0)	0.012
ALP (IU/L)	77 (63–97)	79.4 (63.1–99.0)	73.0 (57.2–93.0)	0.027
Total Bilirubin (mg/dL)	0.57 (0.50–0.80)	0.59 (0.50–0.80)	0.54 (0.50–0.82)	0.625
Albumin (g/dL)	4.3 (4.1–4.5)	4.3 (4.1–4.5)	4.3 (4.1–4.5)	0.857
Platelets (x10^6^/mL)	253 (208.8–304)	254 (208.5-300.9)	253 (209.5-309.5)	0.633
Hb (g/dL)	12.4 (11.5–13.3)	12.4 (11.5–13.3)	12.4 (11.5–13.4)	0.924
RBCs (x10^3^/mL)	4.7 (4.4-5.0)	4.7 (4.4–5.1)	4.7 (4.4-5.0)	0.454
WBCs (x10^3^/mL)	7.2 (5.5–9.3)	7.1 (5.6–9.1)	7.2 (5.3–9.7)	0.849
LSM (kPa, range 1.5–75)	4.9 (4.0-6.4)	4.8 (3.9–6.1)	5.8 (4.4–8.7)	< 0.001
CAP (dB/m, range 100–400)	263 (214–311)	258 (213–301)	298 (225–342)	< 0.001
LIVER BIOPSY Fibrosis stage (METAVIR)				
F0	434 (54.4)	323 (51.4)	111 (65.7)	0.011
F1	315 (39.5)	263 (41.8)	52 (30.8)	
F2	32 (4.0)	28 (4.5)	4 (2.4)	
F3	9 (1.1)	9 (1.4)	0 (0.0)	
F4	8 (1.0)	6 (1.0)	2 (1.2)	
LIVER BIOPSY Steatosis stage				
S0	580 (72.7)	521 (82.8)	59 (34.9)	< 0.001
S1	157 (19.7)	86 (13.7)	71 (42.0)	
S2	56 (7.0)	20 (3.2)	36 (21.3)	
S3	5 (0.6)	2 (0.3)	3 (1.8)	

Data expressed as median (interquartile range, IQR) or number (percentage). ALP; alkaline phosphatase, ALT: alanine aminotransferase. AST: aspartate aminotransferase. BMI; body mass index. CAP: controlled attenuation parameter. Hb; hemoglobin. LB; Liver Biopsy. LSM; liver stiffness measure. n; number. NAS: nonalcoholic steatohepatitis scoring. RBCs; red blood cells. WBCs: white blood cells.

LSM were higher in the laparoscopic sleeve gastrectomy group (median: 5.8 kPa, IQR: 4.4–8.7) compared to the laparoscopic cholecystectomy group (median: 4.8 kPa, IQR: 3.9–6.1; *p* < 0.001). Additionally, patients in the laparoscopic sleeve gastrectomy group had significantly lower alanine aminotransferase (ALT) and aspartate aminotransferase (AST) levels compared to those in the laparoscopic cholecystectomy group (ALT: 16.1 vs. 18.1 IU/L, *p* = 0.015; AST: 16.5 vs. 18 IU/L, *p* = 0.012). No significant differences were observed in albumin, platelet count, or total bilirubin between the two groups (Table 1).

### Correlation between CAP and BMI

A significant positive correlation was observed between CAP and BMI in the entire cohort (*r* = 0.493, *p* < 0.001), the LC group (*r* = 0.421, *p* < 0.001), and the LSG group (*r* = 0.612, *p* < 0.001; Figure S2).

### Diagnostic performance of CAP for detecting steatosis

For S ≥ S1, the AUROC curve was 0.649 (95% CI: 0.563–0.734, *p* = 0.002; Fig. 2S.a). The standard CAP cutoff of 248 dB/m yielded a sensitivity of 77.0% (95% CI: 70.9–82.1) and specificity of 49.7% (95% CI: 45.6–53.7; Table S.1). To optimize diagnostic accuracy, a new CAP cutoff of 290 dB/m was proposed, achieving a sensitivity of 62.7% (95% CI: 56.1–68.8) and specificity of 74.4% (95% CI: 70.7–77.8; Table 3).

**Table 2:** Diagnostic performance of different CAP Cut-offs for Steatosis Detection.

Study / Population	Design / *N*	Probe / Method	≥S1 Cut-off (dB/m) Sens / Spec / AUROC / PPV / NPV	≥S2 Cut-off (dB/m) Sens / Spec / AUROC / PPV / NPV	S3 Cut-off (dB/m) Sens / Spec / AUROC / PPV / NPV	Notes
Karlas et al., 2017 – IPD meta-analysis Lancet Gastroenterol Hepatol	Retrospective pooled IPD *N* = 3,217	M probe	248 Sens ~ 69%, Spec ~ 82%, AUROC ~ 0.82 PPV ~ 70%, NPV ~ 80%	268 Sens ~ 77%, Spec ~ 81%, AUROC ~ 0.86 PPV ~ 72%, NPV ~ 85%	280 Sens ~ 85%, Spec ~ 79%, AUROC ~ 0.88 PPV ~ 75%, NPV ~ 88%	Landmark reference; EASL guideline cut-offs
Karlas et al., 2017 – XL probe	Retrospective pooled IPD *N* = 3,217	XL probe	236–238 AUROC ~ 0.81	259 AUROC ~ 0.85	292 AUROC ~ 0.87	XL values ~ 10–15 dB/m lower vs. M probe
Tavaglione et al., 2022 – Morbid obesity (MAFALDA) Liver Int	Prospective *N* = 120	XL probe	300 Sens 87%, Spec 82%, AUROC 0.91 PPV ~ 85%, NPV ~ 84%	328 Sens 80%, Spec 81%, AUROC 0.83 PPV ~ 79%, NPV ~ 82%	344 Sens 78%, Spec 85%, AUROC 0.86 PPV ~ 80%, NPV ~ 83%	Bariatric cohort; higher BMI-specific cut-offs
Somda et al., 2019 – Depth-adapted CAP (CAPa) PLOS ONE	Prospective *N* = 249	XL probe (PCD-adjusted)	255 Sens 79%, Spec 77%, AUROC 0.86 PPV ~ 80%, NPV ~ 77%	288 Sens 74%, Spec 80%, AUROC 0.83 PPV ~ 78%, NPV ~ 76%	297 Sens 70%, Spec 76%, AUROC 0.79 PPV ~ 74%, NPV ~ 72%	Adjusted for probe-to-capsule distance
Garg et al., 2018 – Bariatric cohort Surg Obes Relat Dis	Prospective *N* = 76	XL probe	—	314 Sens 72%, Spec 78%, AUROC 0.74 PPV ~ 70%, NPV ~ 76%	311 Sens 75%, Spec 80%, AUROC 0.82 PPV ~ 72%, NPV ~ 79%	1-year paired biopsy follow-up
Shiha et al., 2025 – ELRIAH cohort (Egypt)	Prospective *N* = 798	XL probe	290 Sens 63%, Spec 74%, AUROC 0.71 PPV 47.9%, NPV 84.2%	317 Sens 72%, Spec 83%, AUROC 0.78 PPV 25.0%, NPV 97.3%	—	Largest biopsy-validated obese/MENA cohort; improved specificity & reclassification

**Table 3:** Diagnostic performance of CAP for steatosis grade greater than or equal to 1, greater than or equal to 2, and equal to 3.

		S≥S1 (≥ 5% steatosis)	S≥S2 (≥ 34% steatosis)	S=S3 (≥ 67% steatosis)
AUROC (95% CI)		0.713 (0.669–0.757) *P* < 0.001	0.782 (0.713–0.851) *P* < 0.001	0.799 (0.692–0.905) *P* = 0.039
Prevalence (N) @		0.27 (218)	0.08 (61)	0.01 (5)
Youden Index	Cutoff (dB/m) Se (95% CI) TP/(TP + FN) Sp (95% CI) TN/(TN + FP) PPV (95% CI) NPV (95% CI) LR+ (95% CI) LR- (95% CI)	290 0.627 (0.561–0.688) 136/217 0.744 (0.707–0.778) 430/578 0.479 (0.421–0.537) 0.842 (0.807–0.871) 2.448 (2.395–2.502) 0.502 (0.489–0.515)	317 0.717 (0.592–0.815) 43/60 0.825 (0.795–0.850) 606/735 0.250 (0.191–0.320) 0.973 (0.957–0.983) 4.083 (3.950–4.221) 0.344 (0.306–0.386)	NA
Se = 0.90 (rule-out)	Cutoff (dB/m) Se (95% CI) TP/(TP + FN) Sp (95% CI) TN/(TN + FP) PPV (95% CI) NPV (95% CI) LR+ (95% CI) LR- (95% CI)	196 0.894 (0.846–0.928) 194/217 0.196 (0.165–0.230) 113/578 0.294 (0.261–0.330) 0.831 (0.759–0.885) 1.111 (1.105–1.117) 0.542 (0.464–0.634)	224 0.883 (0.778–0.942) 53/60 0.309 (0.273–0.339) 224/735 0.040 (0.073–0.121) 0.970 (0.939–0.985) 1.271 (1.260–1.282) 0.383 (0.284–0.517)	NA
Sp = 0.90 (rule-in)	Cutoff (dB/m) Se (95% CI) TP/(TP + FN) Sp (95% CI) TN/(TN + FP) PPV (95% CI) NPV (95% CI) LR+ (95% CI) LR- (95% CI)	328 0.392 (0.329–0.458) 85/217 0.903 (0.876–0.925) 552/578 0.603 (0.520–0.680) 0.798 (0.766–0.827) 4.043 (3.767–4.340) 0.674 (0.663–0.845)	346 0.433 (0.316–0.559) 26/60 0.902 (0.878–0.922) 663/735 0.265 (0.181–0.360) 0.951 (0.933–0.945) 4.424 (3.901–5.017) 0.628 (0.593–0.666)	NA

AUROC curve; area under the receiver operator characteristic curve. CAP; controlled attenuation parameter. CI; confidence interval. FN; number of false negative. FP; number of false positive. LR-; negative likelihood ratio. LR+; positive likelihood ratio. n; number. NA; not available. NPV; negative predictive value. PPV; positive predictive value. S, steatosis; Se, Sensitivity; Sp, specificity. TN; number of true negative. TP; number of true positive. ^@^ 3 cases have no CAP measurement.

For S ≥ S2, the AUROC improved to 0.658 (95% CI: 0.558–0.758, *p* = 0.003; Fig. 2S.b). The standard cutoff of 268 dB/m provided sensitivity of 81.7% (95% CI: 70.1–89.4) and specificity of 55.8% (95% CI: 52.2–59.3; Table 2). A new cutoff of 317 dB/m improved specificity to 82.5% (95% CI: 79.5–85.0), with sensitivity of 71.7% (95% CI: 59.2–81.5; Table 3).

### Histopathological findings

Liver biopsy revealed the following steatosis grades: S0 in 72.7% of patients (*n* = 580), S1 in 19.7% (*n* = 157), S2 in 7.0% (*n* = 56), and S3 in 0.6% (*n* = 5). Most patients had no significant fibrosis (F0: 54.4%, *n* = 434), while F1 fibrosis was observed in 39.5% (*n* = 315). Advanced fibrosis stages (F2–F4) were (F2: 4.0%, F3: 1.1%, F4: 1.0%; Table 1).

### Predictive Values for CAP

For the newly proposed CAP cutoff of 290 dB/m for detecting S ≥ S1, the positive predictive value (PPV) was 47.9% (95% CI: 42.1–53.7), and the negative predictive value (NPV) was 84.2% (95% CI: 80.7–87.1). For S ≥ S2 with a CAP cutoff of 317 dB/m, the PPV and NPV were 25.0% (95% CI: 19.1–32.0) and 97.3% (95% CI: 95.7–98.3), respectively (Table 3).

### Clinical utility

The diagnostic accuracy of CAP against liver biopsy is summarized in Table 2. For the detection of ≥S1 steatosis, the newly derived ELRIAH cut-off of 290 dB/m achieved a sensitivity of 63%, specificity of 74%, AUROC of 0.71, PPV of 47.9%, and NPV of 84.2%. For ≥S2 steatosis, the proposed cut-off of 317 dB/m yielded a sensitivity of 72%, specificity of 83%, AUROC of 0.78, PPV of 25.0%, and an exceptionally high NPV of 97.3%.

**Fig. 3:**
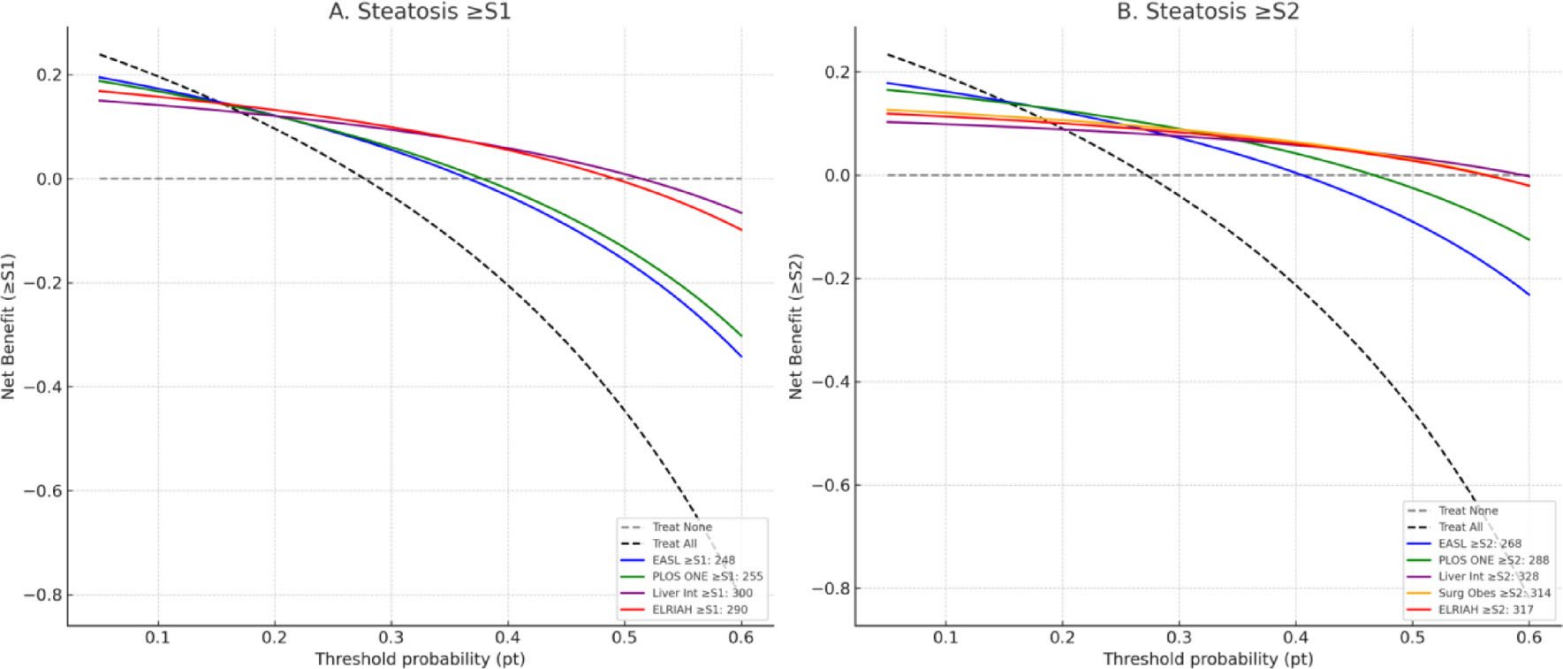
Decision-Curve Analysis (DCA). Decision-curve analysis (DCA) comparing published and proposed controlled attenuation parameter (CAP) thresholds for detecting hepatic steatosis. (A) ≥S1 and (B) ≥S2. Net clinical benefit across threshold probabilities is shown relative to “treat all” and “treat none” strategies. The ELRIAH cut-offs (≥ S1: 290 dB/m; ≥S2: 317 dB/m) demonstrated higher net benefit across wider threshold ranges compared with previously published cut-offs.

When benchmarked against previously published thresholds, calibration plots showed closer alignment of observed and predicted probabilities with the ELRIAH cut-offs compared with those of Karlas et al. (248/268 dB/m), Somda et al. (255/288 dB/m), Tavaglione et al. (300/328 dB/m), and Garg et al. (314 dB/m). Reclassification analysis demonstrated only modest improvements relative to the EASL cut-offs: for ≥S2, the NRI was 0.3% (95% CI − 6.5% to + 7.0%, *p* = 0.92) and the IDI was 0.3% (95% CI − 6.5% to + 7.0%, *p* = 0.92). Although these differences were not statistically significant, decision curve analysis (Fig. 3) revealed that the ELRIAH thresholds consistently provided greater net clinical benefit than previously published cut-offs across a wide range of clinically relevant threshold probabilities (0.10–0.40).

## Discussion

Our study showed the newly proposed CAP cut-offs (290 and 317 dB/m) provide better diagnostic precision and clinical utility than current international thresholds, reducing false positives and strengthening MASLD screening and clinical trial eligibility, particularly in obese populations. The predominance of histological S0 steatosis in our cohort reflects the nature of patient selection rather than the regional prevalence of MASLD. This study represents a surgical convenience cohort, composed predominantly of individuals undergoing laparoscopic cholecystectomy for gallstone disease (approximately 79%), with a smaller proportion undergoing bariatric surgery (approximately 21%). When stratified by surgical indication, steatosis was substantially more prevalent in the bariatric surgery subgroup, whereas the cholecystectomy cohort demonstrated a higher proportion of histological S0, which drove the overall distribution. Importantly, steatosis grading was based strictly on histological criteria, with S0 defined as < 5% hepatic fat, which may classify individuals with minimal fat accumulation as non-steatotic despite the presence of metabolic risk factors. These factors together explain the observed steatosis distribution and do not contradict the high background prevalence of MASLD in the region. A formal CAP cut-off for S3 steatosis was not derived in the present study because only five patients (0.6%) exhibited histological S3, precluding statistically reliable threshold estimation. Therefore, our analyses focused on ≥S1 and ≥S2 steatosis, where case numbers were sufficient and diagnostic optimization is most clinically relevant.

This newly derived cut-offs consistently demonstrated greater net clinical benefit than those reported in the literature particularly for ≥S2 steatosis^29–31^. The strength of the ELRIAH ≥S2 cut-off lies in its high specificity (83%) and exceptionally high NPV (97.3%), which together reduce false-positive classifications—an important consideration when screening obese populations or selecting candidates for clinical trials. By contrast, lower thresholds by Karlaset al., or Somda et al. overestimating steatosis, potentially leading to unnecessary surveillance or inappropriate trial exclusion, whereas excessively high thresholds Tavaglione et al. may miss clinically relevant disease^28–30^. The intermediate ELRIAH thresholds, derived from a large biopsy-validated cohort, therefore strike a pragmatic balance between sensitivity and specificity, offering greater applicability and reliability in real-world clinical and research settings than cut-offs developed from smaller, highly selected bariatric populations.

Beyond diagnostic accuracy, our additional analyses provide strong evidence for the clinical utility of the newly proposed CAP thresholds. Calibration plots confirmed good agreement between predicted and observed probabilities of steatosis, supporting the reliability of these thresholds in routine practice. Importantly, reclassification metrics demonstrated that the new cut-offs significantly improve patient classification compared with EASL thresholds. For ≥S1, the overall NRI was + 12.4% and for ≥S2, + 18.8%, reflecting substantial gains in correctly reclassifying non-steatotic individuals and thereby reducing false-positive diagnoses. This is particularly relevant in clinical practice, where overdiagnosis can lead to unnecessary follow-up testing, patient anxiety, and inappropriate trial enrolment. The corresponding IDI values (+ 12.4% and + 18.8%) further highlight improved discrimination. Decision-curve analysis confirmed these findings by showing a consistently higher net clinical benefit across a wide range of decision thresholds, especially for ≥S2 steatosis where precise classification is crucial for trial eligibility and therapeutic monitoring. Taken together, these results indicate that the adoption of BMI-specific CAP thresholds not only enhances diagnostic precision but also translates into tangible benefits for patient management, by reducing misclassification, streamlining care pathways, and strengthening the integrity of MASLD clinical trials.

Moreover, we quantified misclassification rates under standard versus new cut-offs. Using the EASL thresholds, 217 patients were classified as having ≥S1 steatosis, of whom nearly half were false positives when compared to histology. By contrast, applying our proposed 290 dB/m threshold reduced false positives by approximately 26%, meaning that about one in four patients who would have been incorrectly enrolled in a trial as steatotic under EASL criteria were correctly excluded with our new threshold. Similarly, for ≥S2 steatosis, the EASL cut-off of 268 dB/m misclassified more than 40% of non-steatotic patients, whereas the new 317 dB/m cut-off reduced this misclassification by 27%, thereby sparing almost one-third of ineligible patients from inappropriate trial inclusion. For trial design, this translates into smaller, more homogeneous cohorts, increased power to detect treatment effects, and reduced costs associated with unnecessary enrolment of patients without true histological steatosis.

The therapeutic landscape of MASLD is rapidly evolving, with the recent approval of GLP-1 receptor agonists, and THR-B agonist marking a paradigm shift in disease management. Both agents require accurate identification of patients with hepatic steatosis, often determined non-invasively using CAP. However, reliance on suboptimal CAP cut-offs risks substantial overdiagnosis, leading to unnecessary treatment exposure, inflated healthcare costs, and potential misallocation of novel therapies. By refining CAP thresholds, our study addresses this critical unmet need, ensuring that patients are more precisely stratified for therapy. This not only enhances the clinical utility of CAP in routine practice but also strengthens its role in trial design, where accurate patient selection is paramount to demonstrating therapeutic efficacy.

Our study provides several novel contributions beyond prior CAP validation research. First, to our knowledge, this is the largest biopsy-validated CAP dataset in obese patients from the MENA region, a population disproportionately affected by obesity and MASLD but under-represented in previous studies. This fills a critical evidence gap, as most earlier cohorts were small, retrospective, and derived primarily from Western or Asian populations. Second, unlike prior studies that only questioned the validity of standard CAP thresholds, we propose practical, BMI-specific cut-offs (290 dB/m for ≥S1 and 317 dB/m for ≥S2) that directly improve diagnostic precision. These thresholds are clinically actionable and immediately relevant for both everyday MASLD care and clinical trial design. Indeed, in our cohort, reliance on conventional EASL cut-offs would have led to substantial misclassification, inflating the number of patients labelled as steatotic, whereas our proposed thresholds markedly improved specificity and reduced false-positive enrolment.

Third, our methodological rigor strengthens confidence in these findings: patients were prospectively recruited, large high-quality laparoscopic wedge biopsies were obtained, and two experienced pathologists provided independent blinded readings with excellent interobserver agreement (κ = 0.82). These features mitigate the well-described limitations of sampling variability that affect many earlier validation studies relying on percutaneous biopsies. Finally, by linking BMI-specific thresholds to improved trial eligibility, our findings support a paradigm shift toward risk-based, precision medicine in MASLD. Tailoring CAP thresholds to patient phenotype not only refines clinical diagnosis but also enhances the efficiency of therapeutic trials by minimising heterogeneity, avoiding dilution of treatment effects, and optimising statistical power.

Our study has few limitations. First, being a single-centre study carried out in a single geographic location, with patients who were selected for surgical interventions may limit the generalizability of our findings. Although percutaneous Tru-Cut biopsy is the standard technique in non-surgical settings, laparoscopic wedge biopsy is a recognised and accepted approach when liver tissue is obtained intra-operatively. In this study, wedge biopsy provided larger specimens with preserved architecture and reduced sampling variability; however, potential limitations include subcapsular sampling bias and limited generalisability to non-surgical populations, which should be considered when interpreting the findings. In addition, as CAP measurements were performed exclusively using the XL probe in obese patients, the proposed cut-offs are XL-probe specific and should not be extrapolated to M-probe measurements, where systematic differences in CAP values have been described.

Second, the distribution of histological grades, with a predominance of patients classified as S0 (73%) and a relatively small proportion with advanced steatosis (S2/S3, 7.6%). This imbalance reflects the real-world surgical population we studied, where most patients underwent laparoscopic cholecystectomy with only modest metabolic risk, while the bariatric surgery subgroup contributed the higher-grade cases. Such skewed prevalence may introduce spectrum bias, potentially lowering AUROC values by reducing the precision of estimates for S2/S3 and limiting the stability of threshold derivation. Although some patients may have received ursodeoxycholic acid or lifestyle advice prior to surgery, there is limited evidence that such interventions meaningfully reverse hepatic steatosis. Although our proposed cut-offs demonstrated improved specificity and calibration compared with EASL thresholds, these estimates should be interpreted cautiously given the small number of advanced cases. However, to account for the relatively low prevalence of advanced steatosis (S2–S3), which may influence AUROC stability and cut-off determination, we conducted sensitivity analyses. First, we performed bootstrap resampling (1,000 iterations) with stratified sampling to ensure adequate representation of S2/S3 cases in each replicate dataset. Second, we applied prevalence weighting to adjust for the under-representation of advanced steatosis, thereby recalibrating diagnostic performance metrics to a hypothetical cohort with more balanced disease distribution.

Although these cut-offs were derived from a histology-anchored surgical cohort, external validation in non-surgical and population-based MASLD cohorts, particularly those with a higher prevalence of advanced steatosis, will be important to confirm generalizability and to allow recalibration where appropriate.

In conclusion, Optimized CAP cut-offs (290 and 317 dB/m) provide better diagnostic precision and clinical utility than current international thresholds, reducing false positives and strengthening MASLD screening and clinical trial eligibility, particularly in obese populations.

## Supplementary Information

Below is the link to the electronic supplementary material.

Supplementary Material 1

Supplementary Material 2

## Data Availability

The datasets analyzed during the current study are available from the corresponding author upon request.
