# Supplementary material for: Optimized CAP cut-offs for metabolic dysfunction associated steatotic liver disease in patients living with obesity: a large biopsy-based prospective study
Source: Sci Rep. 2026 Apr 20;16:12894. doi: 10.1038/s41598-026-47209-y (PMC13096376; doi:10.1038/s41598-026-47209-y)
Supplement: Supplementary file 1 — Supplementary Material 1 [file 41598_2026_47209_MOESM1_ESM.docx]

**Figure S1:**

Flowchart of patients included in the study. Of 1127 patients enrolled, 936 had the FibroScan examination performed and 904 had a valid FibroScan examination. Eventually 798 had valid FibroScan measurements and steatosis and fibrosis grades assessed on Liver biopsy.

**Figure S2:**

**2a:** Diagnostic performance of controlled attenuation parameter (CAP) for steatosis grade greater than or equal to S1 (AUROC curve 0.649; P=0.002 and 95% CI: 0.563-0.734). **2b:** Diagnostic performance of controlled attenuation parameter (CAP) for steatosis grade greater than or equal to S2 (AUROC curve 0.658; P=0.003 and 95% CI: 0.558-0.758).

**Figure S3:**

Boxplot of (A) CAP *versus* steatosis grade, CAP values increase with increasing steatosis grade (Kruskal-Wallis test P<0.001). (B) LSM *versus* fibrosis stage, LSM values increase with increasing fibrosis stage (Kruskal-Wallis P=0.012).

## Figures:


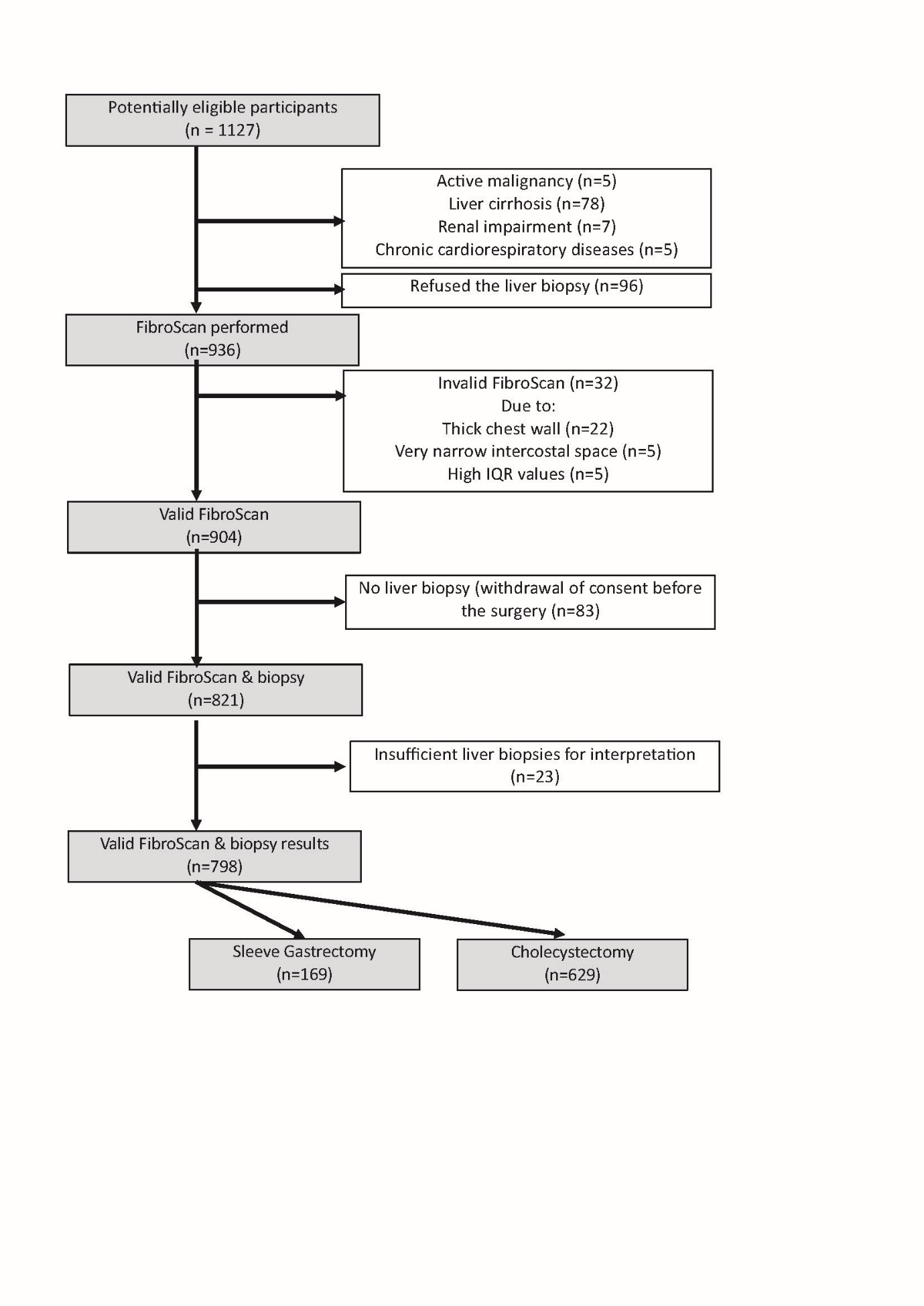


**Figure S1:**

# A

#
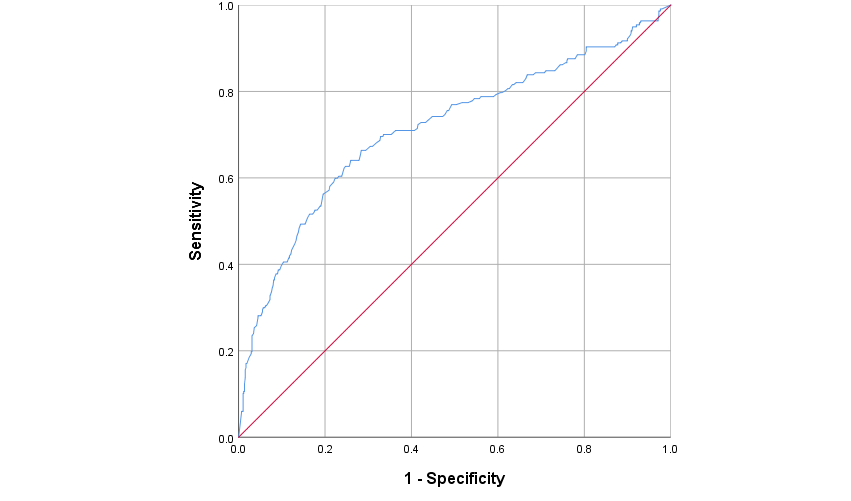


# B
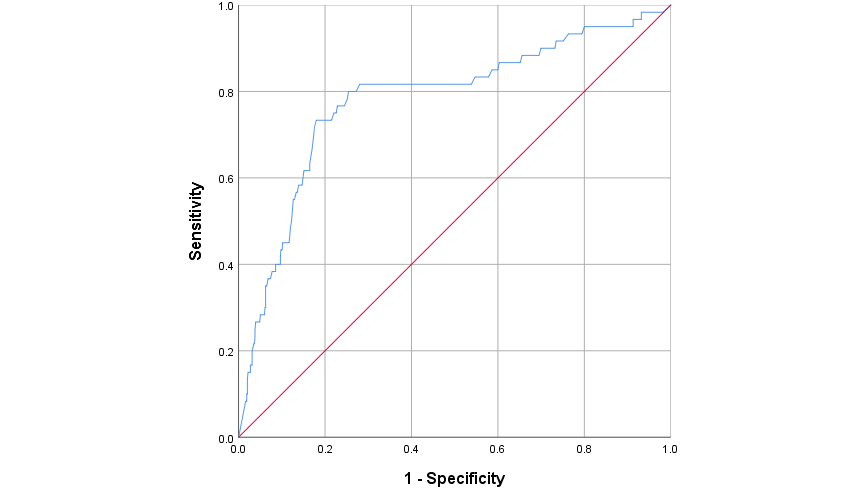


# Figure S2:

| **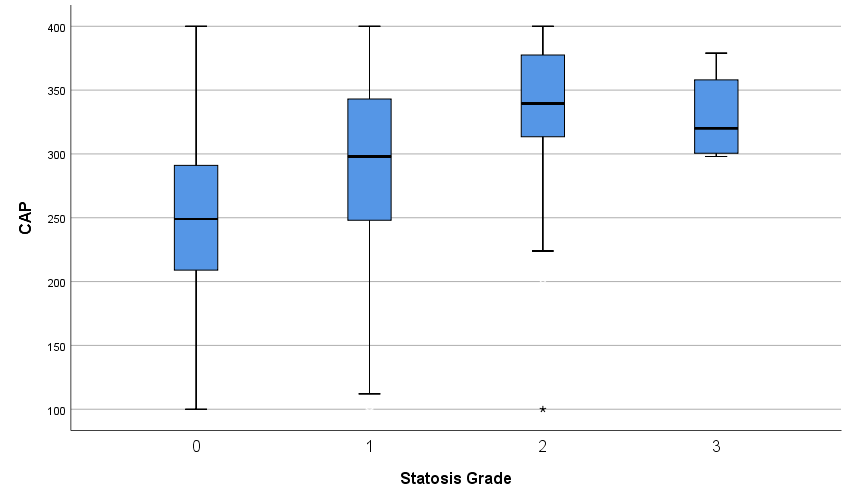** |
| --- |
|  |
| **Figure S3:** |
|  |
