# Supplementary material for: Optimized CAP cut-offs for metabolic dysfunction associated steatotic liver disease in patients living with obesity: a large biopsy-based prospective study
Source: Sci Rep. 2026 Apr 20;16:12894. doi: 10.1038/s41598-026-47209-y (PMC13096376; doi:10.1038/s41598-026-47209-y)
Supplement: Supplementary file 2 — Supplementary Material 2 [file 41598_2026_47209_MOESM2_ESM.docx]

Table S.1: Diagnostic performance of CAP for steatosis grades depending on current cut-off values^#^

| **Index** | **Steatosis (s) ≥ S1**  **(≥5% steatosis)** | **Steatosis (s) ≥ S2**  **(≥34% steatosis)** |
| --- | --- | --- |
| Cutoff ^#^ | 248 | 268 |
| Se (95% CI) | 0.770 (0.709-0.821) | 0.817 (0.701-0.894) |
| TP/(TP+FN) | 167/217 | 49/60 |
| Sp (95% CI) | 0.497 (0.456-0.537) | 0.558 (0.522-0.593) |
| TN/(TN+FP) | 287/578 | 410/735 |
| PPV (95% CI) | 0.365 (0.322-0.410) | 0.131 (0.10.1-0.169) |
| NPV (95% CI) | 0.852 (0.810-0.886) | 0.974 (0.954-0.985) |
| LR+ (95% CI) | 1.529 (1.513-1.544) | 1.847 (1.819-1.875) |
| LR- (95% CI) | 0.464 (0.443-0.486) | 0.329 (0.274-0.394) |
| Diagnostic Accuracy | 0.571 (0.536-0.605) | 0.577 (0.543-0.611) |

*^#^ Source: Karlas et al., 2017.^25^ CAP; controlled attenuation parameter. CI; confidence interval. FN; number of false negative. FP; number of false positive. LR-; negative likelihood ratio. LR+; positive likelihood ratio. n; number. NPV; negative predictive value. PPV; positive predictive value. S, steatosis; Se, Sensitivity; Sp, specificity. TN; number of true negative. TP; number of true positive.*
